# von Willebrand factor activity and activated partial thromboplastin time as proxy biomarkers for coagulopathies in women with menorrhagia in Zambia: a case-control study

**DOI:** 10.11604/pamj.2021.39.13.13742

**Published:** 2021-05-05

**Authors:** Miyoba Melinda Munsanje, Trevor Kaile, Sumbukeni Kowa, Musalula Sinkala, Marah Simakando, Jacob Ndhlovu, Brian Chanda Chiluba

**Affiliations:** 1 Lusaka Apex Medical University, Faculty of Medicine, Department of Pathology, Lusaka, Zambia,; 2 University of Zambia, School of Medicine, Department of Biomedical Sciences, Lusaka, Zambia,; 3 University of Zambia, School of Medicine, Lusaka, Zambia,; 4 University Teaching Hospital, Foods and Drugs Laboratory, Lusaka, Zambia,; 5 Eden University, Faculty of Medicine, Lusaka, Zambia,; 6 University of Zambia, School of Public Health, Lusaka, Zambia

**Keywords:** Activated partial thromboplastin time, bleeding disorders, blood groups, menorrhagia, von Willebrand factor activity, von Willebrand disease

## Abstract

**Introduction:**

von Willebrand Disease (vWD) is the most prevalent bleeding disorder. Women are more likely to manifest abnormal bleeding symptoms due to physiologic events and menorrhagia is the most common presenting symptom.

**Methods:**

this case-control study included 168 women aged between 18 and 45. The cases had menorrhagia whilst the controls did not. Blood grouping, activated partial thromboplastin time and von Willebrand factor activity tests were performed on samples collected from consenting study participants.

**Results:**

the mean age was 29.96 ± 7.37. Mean vWF activity of cases was 66.6% and of controls 97.8%. The mean activated Partial ThromboplastinTime (aPTT) of cases was 31.09s and of controls was 30.40s. There was no difference in the vWF activity between blood group O (86.3%) and non-blood group O (88.0%) participants. Eight women were diagnosed with von Willebrand disease, 6 cases and 2 controls. Higher odds of von Willebrand disease were seen in the cases (OR = 6.6). Epistaxis, von Willebrand and factor activity levels and family history of menorrhagia were associated with an increased risk for menorrhagia.

**Conclusion:**

von Willebrand factor activity levels were associated with menorrhagia while activated partial thromboplastin time was not. vWF activity levels did not depend on any specific blood group. The prevalence of von Willebrand disease was significantly higher in participants with menorrhagia and repeated epistaxis and family history of menorrhagia pointed to a higher risk of menorrhagia.

## Introduction

von Willebrand disease (vWD) is a common, inherited, genetically and clinically heterogeneous hemorrhagic disorder caused by a deficiency or dysfunction of the protein von Willebrand factor (vWF) [1]. von Willebrand Disease (vWD) is usually autosomal dominant and results from mutations in the von Willebrand factor gene. This results in a deficiency or functional abnormality of von Willebrand Factor (vWF) which is a glycoprotein that acts as a carrier protein for factor VIII (anti haemophilic factor) and as an adhesive protein in platelet-vessel wall interactions [1,2]. It is the most prevalent inherited bleeding disorder worldwide, affecting 1 to 3 percent of the world population [3].

Individuals with vWD are at an increased risk for muco-cutaneous bleeding that includes epistaxis, easy bruising, prolonged bleeding after trivial cuts, excessive bleeding with dental procedures, excessive bleeding from the oral cavity, gastrointestinal bleeding, excessive post-operative bleeding, and reproductive tract bleeding. Clinical features of vWD include bleeding typically from membranes, (mouth, epistaxes, menorrhagia, and excess blood loss during trauma or surgery. Diagnosis includes prolonged aPTT, Factor VIII and von Willebrand Factor (vWF) levels are low with a prolonged bleeding time. von Willebrand Factor activity measures the activity of vWF. It is expressed as percentage of the normal. Activated partial thromboplastin time is a laboratory based test for haemostasis. It measures the intrinsic and common pathway of coagulation. It is prolonged in von Willebrand disease and is used as one of a panel of tests for diagnosis of vWD. Normal ranges are 30 to 40 seconds [1]. Even though vWD affects males and females equally, females are more likely to present with symptoms due to physiologic events like menstruation, child birth, surgery involving child birth and others. Most studies have used menorrhagia as an indicator for bleeding disorders because it is the most common symptom in women with a disorder and could even be the only presenting symptom [4].

The most common symptom of vWD in women is menorrhagia. Menorrhagia is defined as greater than 80 cubic centimeters (cc) of blood loss per cycle which is the amount of blood loss that would result in iron deficiency anaemia. The following symptoms and findings are associated with menorrhagia and include: soaking through a pad or tampon within 1 hour, soaking through bed clothes, below normal ferritin, anaemia, and a pictorial blood assessment chart score of greater than 100. Additional bleeding symptoms include epistaxis, bleeding after dental extraction, ecchymoses, bleeding from minor cuts or abrasions, gingival bleeding, haemarthrosis and gastrointestinal bleeding. A number of studies have evaluated the presence of bleeding disorders in women with menorrhagia. Findings vary among studies and ranges for vWD in women with menorrhagia are 5-20% [5]. People with blood group O have lower amounts of vWF than people with blood groups A, B and AB grouped as non-blood group O. Pathological values for vWF for blood group O people are thus lower than non-blood group O pathological vWF levels. This is due to a shorter survival or elimination half-life in plasma of vWF in blood group O compared to the other blood groups [6,7].

There are no recorded studies of vWD in Zambia. Despite being the most common inherited bleeding disorder is not a routine test for patients who visit the hospital with bleeding abnormalities. The study aimed to determine the levels of von Willebrand factor activity and activated partial thromboplastin time in women with menorrhagia and estimate the prevalence of von Willebrand disease in these women. More specifically, we aimed, firstly, to determine and compare vWF activity and a PTT in women with menorrhagia, the “cases” and women without menorrhagia, the “controls”. Secondly, to evaluate the association between vWF activity and blood groups O verses blood groups A, B and AB in both cases and controls and finally to estimate the prevalence of von Willebrand disease based on vWF activity and aPTT in cases and controls.

## Methods

The research proposal was submitted to the University of Zambia Biomedical Research Ethics Committee (UNZABREC) for approval and was approved on the 8^th^ of April, 2016 with Ref. No. 004-01-16. The study participants were recruited to the study as they came to the Facility for medical attention. The women identified as suitable participants were presented with an information sheet which was available in both English and a vernacular language Nyanja, which is widely spoken in Lusaka, where the study was being conducted. This study was an unmatched case-control study. It included women aged 18 to 45 who visited the Gynaecology and Obstetrics´ (Ob/Gyn) clinic of the UTH from May 2016 to December 2016. The sample size was calculated using an online sample size calculator for an unmatched case-control study using open epi statistical software using a study power of 80%. Fifty-six (56) women with menorrhagia were identified as Cases and 112 women without menorrhagia or any bleeding problems were identified as controls. Informed consent was obtained from participants who signed an informed consent form, which also had a vernacular translation, after reading the information sheet. Consenting women were presented with a questionnaire to fill, this questionnaire was also available in both English and vernacular. The questionnaire contained data on bleeding patterns and family history. Four (4) mls of blood was then collected from participants in sodium citrate containers. The samples were centrifuged for 15 minutes at 1500 rotations per minute and the activated partial thromboplastin time was run on the samples. The plasma was then separated from red blood cells and stored in crial vials at -20 degrees celsius until von Willebrand tests were run. The red cells were used for blood grouping.

**Study subject´s inclusion criteria:** women between the ages of 18 and 45 were included in the study grouped as cases and controls. The cases were women who came to the facility with a complaint of prolonged and heavy menstruation. The controls were women who came to the facility for reasons other than menorrhagia, these were randomly selected. Menorrhagia was self-reported by participants (cases) and diagnosis was based on a definition of prolonged and heavy menstruation lasting seven days or more.

**Study subject´s exclusion criteria:** potential participants were excluded if they were below the age of eighteen (18) and above the age of forty-five (45). For the cases, the women were excluded from the study if they had menorrhagia but the aetiology of the menorrhagia was known such as uterine fibroids and intra uterine devices as a means of birth control.

**Laboratory tests:** three tests namely, activated Partial Thromboplastin Time (aPTT), blood grouping and finally von Willebrand factor activity were analysed on the blood that was collected from the study participants. The blood was collected in sodium citrate tubes. The activated Partial Thromboplastin Time (aPTT) was run within eight hours of sample collection. The samples were transported to the laboratory after collection were they were centrifuged in a centrifuge for 15 minutes at 1500 rotations per minute (rpm). The samples were then separated into plasma and red blood cells. Blood grouping was done using forward grouping methods using commercial anti-sera. The plasma was stored at -20 degrees celsius for von Willebrand factor testing. vWF activity and a PTT tests were run on the C1500 Siemens coagulation machine. For a PTT we used dade actin FS activated PTT reagent. The stored plasma was thawed at room temperature in preparation for vWF activity testing. For vWF activity we made use of innovance vWF Ac, a particle enhanced assay for the automated determination of vWF activity in human citrated plasma. Results were reported as percentage of the normal and were blood group specific.

**Statistical analysis:** all data processing was performed using a commercial software package MATLAB version R2016b (MathWorks Inc, Natick, MA, USA). The Shapiro-Wilk test was used to check for normality, and histograms and box plots were examined to verify the normality of distribution of measurement data, with non-normally distribution data, was transformed using the box-cox transformation. The independent sample t-test was used to evaluate the mean difference of vWF activity and aPTT between women with and without menorrhagia, and of vWF activity between blood group O and non-blood group O women. A one-way ANOVA was used to evaluate mean differences of vWF activity and aPTT in participants with varying menses flow rate. Furthermore, the Chi-squared test or Fisher's exact test where appropriate were used to assess associations between women with a family history of menorrhagia with either in menses flow rate or vWF activity, and menorrhagia with nosebleeds. All statistical tests were performed at the 95% significance level, and differences were considered.

## Results

**Figure 1:**
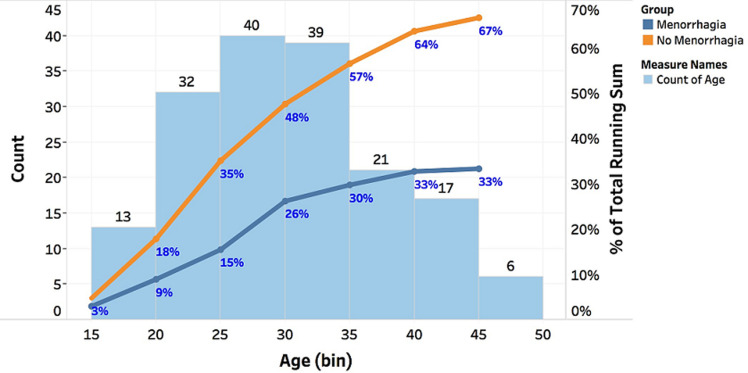
the trends of the age of participants (first y-axis) and percentage of the total running sum of age (second y-axis) for age (bin) on the x-axis; the histogram shows counts for each bin of age; for pane percentage of the total running sum of age: colour shows details about the group

**Figure 2:**
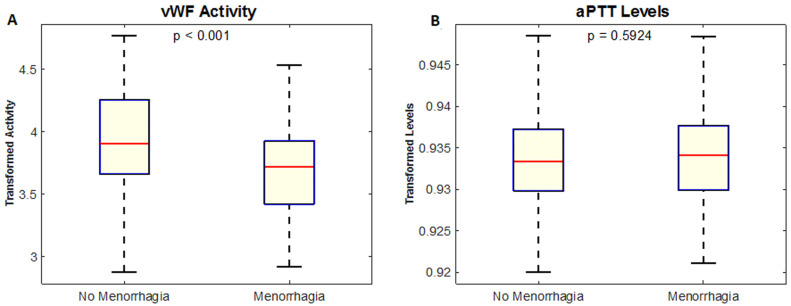
(A,B) the mean VWF activity in women with menorrhagia was lower (66.6%) than that of women without menorrhagia (97.8%) p<0.001; this may explain why these women had menorrhagia; the mean aPTT of the women with menorrhagia was only slightly but not significantly higher (31.09s) than that of women without menorrhagia (30.40s) p = 0.593

**Figure 3:**
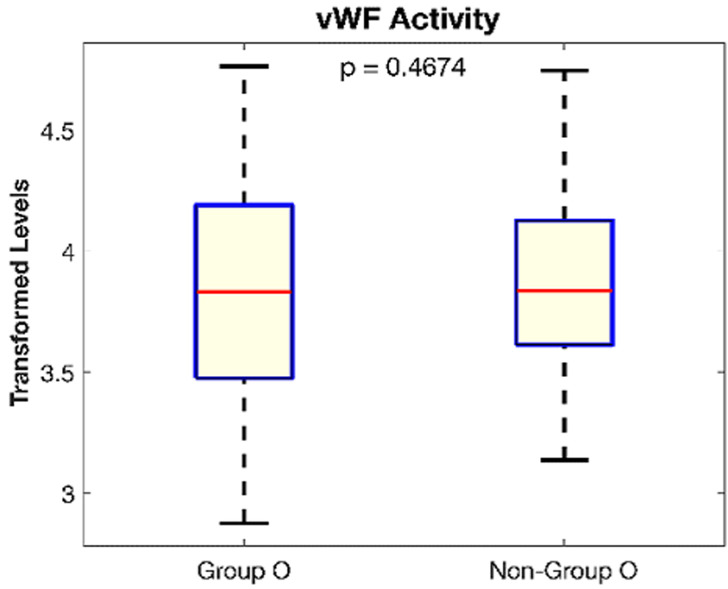
the mean Von Willebrand factor activity levels in Blood group O women was slightly but not significantly lower (86.3%) than in the other women with blood groups A, B and AB (88.0%) p = 0.467

A total of 168 women participated in the study, mean age 29.96±7.37 (range 18 to 45 years) (Figure 1). Our study found that women with menorrhagia had a significantly lower mean vWF activity (66.6±31.5) percent than women without menorrhagia (97.8±53.1) percent, p<0.001 shown in Figure 2, Table 1. We evaluated variations of vWF activity across blood groups, blood group O and non-blood group O (A, B and AB). Blood group participants had a mean vWF activity of 86.3± 51.3 percent while non-blood group O had a mean vWF activity of 88.0 ± 47.5. The difference in means was not significant as assessed by t-test p=0.467 (Figure 3). There was also no association between blood groups and laboratory levels of vWF activity, low, normal and high, p=0.213. The Annova test was conducted to compare vWF activity across participant reported flow rates, low, medium, heavy, very heavy. We found no significant difference among the flow rates, p=0.08.

**Table 1:** descriptive statistics of vWF activity and aPTT levels

Measurements	Condition	N	Mean	Median	Std. Dev.	Range	Min	Max
**vWF activity**	No menorrhagia	112	97.8	80.4	231.7	53.1	22	253.7
	Menorrhagia	56	66.7	63.3	162.5	31.5	23.1	185.6
**aPTT levels**	No Menorrhagia	112	30.4	28.7	42.8	7.7	18.3	61.1
	Menorrhagia	56	31.1	29.5	40.7	8.3	20	60.7

Mean vWF activity was higher in cases than controls. Mean aPTT was higher in cases than controls

**Figure 4:**
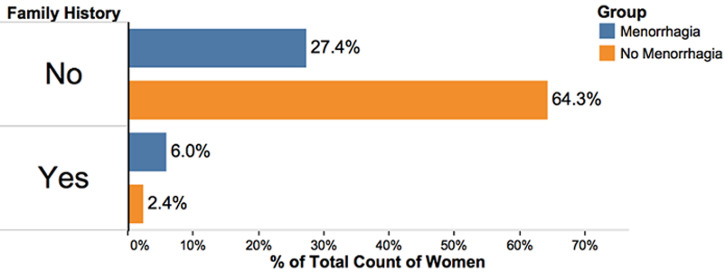
there was a significantly higher percentage of women with menorrhagia (6%) that reported a family history of menorrhagia compared to women without menorrhagia (2.4%) p = 0.003 meaning a positive family history of menorrhagia increased the chances of having menorrhagia

**Figure 5:**
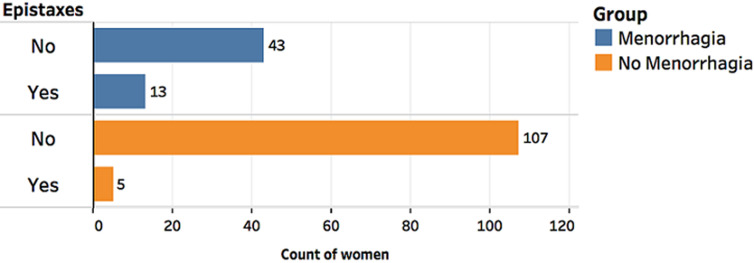
a higher number of women with menorrhagia (13) reported to have suffered epistaxis compared to women without menorrhagia (5); there was a positive association between the menorrhagia and epistaxis p<0.001

Comparison of aPTT Means between the menorrhagia (31.092±8.256s) and non-menorrhagia group (30.39±7.73s) showed no significant difference, p=0.592 (Figure 1). We did not find any difference in aPTT values across participant reported flow rates, p=0.267 (Figure 2, Table 1). Based on both pathological aPTT and vWF activity values, we diagnosed vWD. We found eight out of the 168 women had vWD. Six of these women had menorrhagia (cases) and two did not have menorrhagia. Fifty percent (50%) of these women were blood group O and the other 50% were blood group A. Two of the women with menorrhagia had a family history of abnormal bleeding while one had suffered previous repeated episodes of epistaxis, easy bruising and prolonged bleeding from wounds. Women with menorrhagia had higher odds of having vWD (OR=6.6) and women with menorrhagia had a significantly higher prevalence of vWD as assessed by Fishers exact test, p= 0.024 (Table 2). A positive family history of menorrhagia also increased the chances of participants having menorrhagia, p=0.003. A family history of menorrhagia did not influence a participants vWF activity, p=0.196 (Figure 4). We also found that women with menorrhagia were more likely to encounter recurrent epistaxis compared to the women that did not have menorrhagia, p<0.001 (Figure 5).

**Table 2:** characteristics of cases and controls

Characteristic	Women with menorrhagia n=56	Women without menorrhagia n=112	P value	Odds Ratio
Epistaxis	13 (23.2%)	5(4.5%)	<0.001	
vWD	6 (10.7%)	2(1.8%)	0.017	6.6
**Blood types**				
	**Group O**	**Non-group O**		
	76	89		
vWF activity	86.3	88	0.467	
**Characteristics of women diagnosed with von Willebrand disease**				
	**Ethnicity**	**Age (Years)**	**Blood group**	**Family history of abnormal bleeding**
Control	African	30	A+	No
Control	African	18	O+	No
Case	African	41	A+	Yes
Case	African	28	O+	No
Case	African	24	O+	No
Case	African	27	A+	No
Case	African	29	A+	Yes
Case	African	27	O+	No

## Discussion

Menorrhagia is a common presenting symptom in women of reproductive age with bleeding disorders. Other common causes include fibroids and some forms of contraceptive such as intrauterine devices (IUD) and Depo Provera in some women [8,9]. In cases where the cause is unknown vWD should be suspected. The frequency of vWD is equal in both males and females but it is more apparent in women due to inevitable occurrences of bleeding during menstruation, child birth and caesarean sections. Menorrhagia is often reported at menarche which is normally between ages 11 and 16 [10]. Our study however, only included women between reproductive ages 18 and 45. As menstruation is thought to be a topic of taboo in the African set-up, a lot of women affected by it would rather not seek medical help early or resort to traditional medicines before finally visiting a Health Centre at a later stage. Nearly 100 percent of the participants were of African ethnicity with only one control being of mixed race ethnicity. vWF levels have been found to be lower in African women than in women of Caucasian, Asian and other race descent. Of the 168 women in our study, we found an overall mean vWF activity of (84.65±68.51) percent, which is within normal expected values of vWF activity. A study by Miller *et al*. found a mean vWF activity of 99 percent in African women, their finding was not statistically different between African and Caucasian women in their study. The cause of the race difference is not known [11]. Our study found that the cases had lower levels of vWF activity compared to the controls (p=0.001) (Figure 2, Table 1). vWF activity is expected to be less in women with vWD and consequently menorrhagia as it is the most common presentation. Therefore, our findings of less vWF activity in women with menorrhagia than those without are consistent with literature and other studies [4,5,8]. In a systematic review, Shankar *et al*. reviewed studies of vWD in women with menorrhagia across the world. Nine out of these eleven studies had used VWF activity as one of the tests for diagnosis of vWD and found that 131 (13%) women out of 988 women included in the study were diagnosed as having vWD [12]. Our study found that 6 (10.71%) women out of 56 women with menorrhagia had vWD indicating significantly higher odds (6.6) of vWD in the menorrhagia group compared to the no-menorrhagia group. vWF activity could be seen reducing from light menstrual flow to very heavy menstrual flow. This observation though not significant explains why women with menorrhagia had more pathologic values of vWF activity just as other studies have suggested low vWF activity in menorrhagia patients and disorders of prolonged bleeding [12].

Activated partial thromboplastin time is a laboratory based test for haemostasis. It is prolonged in vWD and is used as one of a panel of tests for diagnosis of vWD. Normal ranges are 30 to 40 seconds [1]. Our cases and controls showed no difference in aPTT scores (p = 0.592) (Figure 2, Table 1). Our findings were in contrast to a study by Mohamedahmed *et al*. who reported a significant difference in aPTT between cases and controls [13]. However, our study showed a decrease in aPTT across self-reported menstrual flows by our study participants from heavy, very heavy, medium and finally light flow had the lowest levels of aPTT which fall in the acceptable range. As some previous studies have reported a significant difference, it would suggest that a prolonged aPTT could be used as a marker for a coagulopathy. A number of studies have confirmed that plasma levels of vWF Antigen and activity are significantly affected by ABO blood group. Blood group O candidates have lower levels of vWF Ag and activity therefore, pathological values for this group are lower than non-blood group O candidates, this difference across blood groups could be due to the shorter vWF survival or elimination half-life in plasma compared to non-blood group O candidates vWF [7,14,15]. It is suggested that this difference across the ABO blood group is due to the presence of blood group antigens on vWF itself, which may affect its interaction with platelet receptors or its clearance from the circulation [7,14,15]. Our study however, was in variance to this hypothesis as we did not find a significant difference between blood group O women and non-blood group O women in terms of vWF activity (p = 0.467) (Figure 3). A much larger sample size may have been required as observed in other studies like Cox JC *et al*. who had 1,117 participants in their study of the effect of ABO blood groups on the diagnosis of vWD which showed that blood group O individuals had the lowest amounts of vWF Ag at 74.8 U/dL. A hypothesis could be tested whether higher amounts of vWF activity were common in Africans [6].

The mean age of women affected with vWD in our study was 28 and 4 (50%) of them were blood group O rhesus positive while the other four (50%) were blood group A rhesus positive. Interestingly our findings were very similar to Dilley *et al*. who also found that the women with vWD were of blood group O and A in almost equal proportions [16]. Out of the six cases, two had a family history of abnormal bleeding while one had suffered previous episodes of bleeding through the nose, easy bruising and prolonged bleeding. We diagnosed vWD based on abnormal vWF activity and an abnormal aPTT. Our study found that 2 controls out 112 (1.78%) had vWD and 6 out of 56 cases (10.71%) had vWD (Figure 4). We found an Odds ratio of 6.6, women with menorrhagia had significantly higher odds of having vWD than women without menorrhagia. Our findings tended to support the observation that menorrhagia was an important indicator for bleeding disorders in women. Our findings are similar to a number of studies that had found a higher prevalence and odds ratio of vWD in women with menorrhagia compared to women without [12]. Dilley *et al*. found a prevalence of 6.6 percent in women with menorrhagia and 0.8 percent in women without menorrhagia and an odds ratio of 8.6 [16]. Most studies previously done, have found higher prevalence´s of vWD, these include studies done in Sudan, Egypt, Iran [17-20]. Finally, a systematic study by Shankar *et al*. concluded that vWD in women with menorrhagia is increased and vWD is a significant cause of menorrhagia in women [12].

Other than comparing the test scores of cases and controls, we found that menorrhagia and epistaxis was significantly associated (p<0.001) (Figure 5). Our findings suggest that women with menorrhagia were more likely to experience epistaxis compared to women who did not suffer from menorrhagia. Similar to our study, Payandeh *et al*. found a significant association between a history of muco-cutaneous bleeding and menorrhagia, nose bleeding was also found to be statistically higher in women who were further diagnosed with vWD [18]. These findings highlighted the need to assess patients with bleeding abnormalities such as menorrhagia for coagulopathies. Our results further suggested that a Physicians´ good patient history taking could assist in the detecting the presence of bleeding disorders in women. This would also prove cost effective for a low income country like Zambia as only selected patients would be considered for coagulopathy testing. Therefore, recurrent Epistaxis could be used as a predictor for coagulation disorders in patients with menorrhagia.

As vWD is genetically inherited, it is expected that people with vWD should have a family history of abnormal bleeding [1]. We found a significant association between participant´s family history of menorrhagia and participant menorrhagia but no significant association between family history of menorrhagia and an abnormal vWF activity. Of a total 168 recruited women 154 (92 percent) had no family history of menorrhagia while 14 (8 percent) did. Similar results were found by Dilley *et al*. who found no significant association between a family history of abnormal bleeding or a disorder and vWD. They also found no significant difference between cases and controls family history of bleeding [16].

## Conclusion

Our study showed that vWF activity was associated with menorrhagia while aPTT was not associated with menorrhagia. Further, vWF activity did not depend on the presence of a specific blood group. Our study also showed that the prevalence of vWD was significantly higher in participants with menorrhagia. Repeated epistaxis and a positive family history of menorrhagia pointed to development of menorrhagia.

### What is known about this topic

- Women with von Willebrand disease have a much higher risk of having menorrhagia compared to women without von Willebrand disease;
- Blood group O people have lower amounts of von Willebrand factor than people of blood groups A, B and AB.

### What this study adds

- Von Willebrand disease can be a cause of menorrhagia in Zambian women with menorrhagia;
- There was no difference between blood group O and non-blood group O von Willebrand factor activity;
- This study may be used as a basis to improve treatment of women with bleeding disorders and help reduce occurrences of post-partum haemmorhage which is one of the leading causes of death in women during and after birth.
